# Feasibility of single-stage direct-to-implant subpectoral breast reconstruction following neoadjuvant chemotherapy for locally advanced breast cancer

**DOI:** 10.1097/MD.0000000000047123

**Published:** 2026-01-30

**Authors:** Berkay Kilic, Burak Ilhan

**Affiliations:** a Department of Surgery, Institute of Oncology, Istanbul University, Istanbul, Turkiye; b Department of Surgery, Istanbul Faculty of Medicine, Istanbul University, Istanbul, Turkiye.

**Keywords:** breast cancer, breast reconstruction, immediate breast reconstruction, oncological safety, recurrence

## Abstract

Breast reconstruction is increasingly requested in breast cancer surgery, particularly among young women. However, the criteria for its feasibility need to be more clearly defined. This study aimed to compare the surgical safety and long-term oncologic survival outcomes between immediate breast reconstruction (IBR) and conventional mastectomy (CMx) and to determine the main factors affecting recurrence, considering tumor-specific features. The study analyzed 297 patients with locally advanced breast cancer (stage IIB–IIIC and/or axilla positive) who underwent CMx or IBR between January 2015 and December 2021 at Istanbul University, Oncology Institute, and mainly investigated the outcomes of both surgical interventions regarding recurrence and survival rates. The study examined 132 patients who underwent the CMx and 165 with the IBR procedure. Median follow-up was 60.2 months (range, 12–107). On average, younger patients (43.36 vs 50.42, *P* < .001) and patients with relatively early-stage tumors (*P* = .007, for cT and *P* = .004, for cN) underwent the IBR procedure more often compared with CMx. There was no difference between the 2 groups regarding other tumor-specific characteristics, receptor profiles, regimens of neoadjuvant chemotherapy, response to neoadjuvant chemotherapy, and adjuvant therapies (*P* > .05 for each comparison). The definitive pT stage had the highest risk for local recurrence, with a 3.2-fold increased risk independent of the type of surgery performed (*P* < .001, 3.22 (1.56–6.66)). However, no difference was between the IBR and CMx groups regarding local recurrence (84.8% vs 85.6%), distant metastasis (81.2% vs 79.5%), free survival rates, and overall survival (94.95 months vs 93.47 months, *P* = .916). Immediate breast reconstruction is a safe and feasible approach in patients with locally advanced breast cancer receiving neoadjuvant therapy compared to CMx alone, as it does not affect recurrence rates and survival.

## 1. Introduction

Locally advanced breast cancer (LABC) is a heterogeneous disease, accounting for approximately 5% to 20% of newly diagnosed breast cancers in developed countries.^[1]^ In contrast, this proportion can reach up to 50% in developing countries, underscoring its significance as a public health issue.^[2,3]^ Currently, neoadjuvant treatment models have become the standard treatment for LABC.^[4]^ Despite therapeutic advancements, however, approximately 40% of patients still undergo conventional mastectomy (CMx).^[5,6]^ To alleviate the psychological and physical side effects of mastectomy, immediate implant-based breast reconstruction is increasingly performed.^[7]^

Immediate implant-based breast reconstruction following nipple-sparing mastectomy (NSM) or skin-sparing mastectomy (SSM) can be performed as either a single-stage or 2-stage procedure.^[8]^ Many surgeons prefer a 2-stage reconstruction in patients with LABC requiring radiotherapy (RT) after mastectomy to minimize complications and prevent delays in adjuvant treatment.^[9]^ Recently, with increasing surgical experience and advancements in mastectomy techniques, there has been a growing shift from 2-stage to single-stage reconstruction. In single-stage breast reconstruction, implants can be placed in either the subcutaneous or the subpectoral plane, making this approach suitable for nearly all patients undergoing NSM or SSM.^[10]^ Acellular dermal matrix (ADM) or mesh is frequently used during the reconstruction to achieve a more natural appearance. A critical consideration is the timely initiation of adjuvant therapy without delay. Previous studies have demonstrated the oncologic safety of both single-stage and 2-stage reconstructions in early-stage breast cancer.^[11,12]^ However, for patients with LABC requiring RT following neoadjuvant chemotherapy (NACT), the safety remains less well-defined. To date, there were no published prospective studies on patients undergoing radiation therapy following single-stage breast reconstruction after NACT.

Despite the limitations associated with being a retrospective study, our research aimed to provide information on the feasibility of immediate breast reconstruction (IBR) as a single-stage direct-to-implant (ss DTI), to share long-term oncological outcomes, and patient satisfaction from both physical and psychological perspectives, as well as the safety of this approach in patients receiving RT after NACT.

## 2. Methods

### 2.1. Study cohort

Breast cancer patients who underwent immediate implant-based breast reconstruction following mastectomy or mastectomy alone at the Breast Center of Istanbul University Oncology Institute between January 1, 2015, and December 31, 2022, were retrospectively analyzed using data from a prospectively maintained database. The analysis included 132 patients who underwent CMx alone and 165 who underwent an IBR. All patients obtained written informed consent. The Ethics Committee of Istanbul University (decision number no: 2024-3024894) approved the study. The study included patients who underwent mastectomy and were clinically diagnosed with LABC at the time of diagnosis. Exclusion criteria included patients who had undergone autologous flap or 2-stage reconstruction, metastatic disease, pathological T4 disease, or those who underwent mastectomy for inflammatory breast cancer, palliative care, or recurrence. Patient follow-up data and oncological outcomes were gathered from the electronic database and medical records. For patients whose information we could not access via a phone connection, we used the data of the most recently reached follow-up date.

A core tissue biopsy confirmed the breast cancer diagnosis. Fine-needle aspiration biopsy under ultrasound guidance diagnosed the spread of suspicious axillary lymph nodes. Tumor staging was determined according to the 8th edition of the American Joint Committee on Cancer staging manual, with breast cancer stages IIB, IIIA, IIIB, and IIIC classified as LABC.^[13]^ Clinical staging involved a physical examination, ultrasonography, mammography, magnetic resonance imaging (MRI), and positron emission tomography-computed tomography at baseline, performed pre- and post-NACT.

The decision to perform mastectomy was contingent upon several factors, including tumor histology, tumor-to-breast size ratio, multifocality/multicentricity, and patient preference. Multifocality was defined as multiple tumor foci within the same quadrant and <5 cm apart, while multicentricity referred to multiple foci in different quadrants. ss DTI IBR was recommended for all patients with an adequate breast volume. Pathologic complete response (pCR) was defined as the absence of residual tumor in both the breast and axilla upon pathologic examination following treatment.

### 2.2. Treatment procedure and follow-up

All patients received NACT with treatment regimens determined according to the guidelines applicable at the time of treatment, as discussed by medical oncologists at the institute’s weekly oncology meeting. The surgery was scheduled between the 4th and 6th week after the completion of chemotherapy. NSM and CMx were the primary surgical techniques. Patients underwent the NSM without preoperative skin or areola involvement or confirmed by intraoperative retroareolar frozen section biopsy. If the biopsy or final pathological examination indicated the presence of a tumor, the procedure necessitated a conversion to SSM. If necessary, patients underwent postoperative nipple and/or areola excision under local anesthesia, without any conversions to CMx. In certain patients with macromastia, free areola grafting or reduction mammoplasty techniques were employed as required.

DTI reconstruction was the preferred technique. Surgeons performed a lateral radial incision from near the nipple-areola complex to the axilla, which was the most commonly used incision. An anterolateral incision was a less preferred option. The commonly selected area for implant placement was the subpectoral plane. In these cases, the lateral area’s partial defect was closed with loose, intermittent sutures connecting the serratus anterior muscle fascia and the pectoral muscle. In this study group, no mesh or biological membranes were utilized. Implant selection was guided by the breast’s anatomical features, utilizing various silicone implant brands and types (Mentor by Johnson & Johnson, USA, and Allergan by AbbVie, USA) in both smooth and textured varieties. For patients with genetic mutations, surgeons performed prophylactic mastectomy of the contralateral breast. Radiation oncologists prescribed adjuvant RT based on pretreatment staging, histological findings, tumor size exceeding 5 cm, and axillary involvement. The radiotherapy fields encompassed the chest wall and regional lymph nodes (infra- and supraclavicular). In patients who underwent IBR, radiotherapy was administered after reconstruction, if deemed appropriate by the radiation oncologists. Postoperatively, patients underwent follow-up examinations every 3 months for the initial 2 years, every 6 months from years 2 to 5, and annually thereafter. Patient records consistently included documentation of complications, re-surgeries, recurrences, and metastases. Pathological confirmation of local or regional recurrences was obtained through biopsy. Positron emission tomography-computed tomography and MRI were employed to primarily assess distant metastases.

### 2.3. Statistical analysis

Continuous variables were presented as median, range, and percentage. Overall survival (OS) was defined as the interval from the commencement of treatment until the time of death or the last follow-up. Disease-free survival (DFS) encompassed the period from the surgical date until the occurrence of recurrence. Local recurrence-free survival (LRFS) denoted the duration from surgery until locoregional recurrence. Distant metastasis-free survival (DMFS) defined the duration from surgery to detecting distant metastasis. Disease-specific survival indicated the period from surgery to death specifically attributed to the disease. The Kaplan–Meier method assessed survival outcomes. Subsequently, a reverse logistic regression model was constructed to identify factors associated with local recurrence. Statistically significant variables with a *P* value <.05 and clinically relevant variables were incorporated into this model to predict the likelihood of local recurrence. The outcomes were presented in tabular format, including mean values and corresponding 95% confidence intervals. Data analysis was performed using Microsoft Excel software (Microsoft Luxembourg S.a.r.l., Luxembourg City, Luxembourg) and IBM SPSS Statistics version 21 (SPSS, Chicago).

## 3. Results

The study analyzed a total of 297 breast cancer patients, with 132 (44.4%) in the CMx group and 165 (55.6%) in the IBR group, all of whom underwent standard ss DTI c. The median follow-up duration was 61 months (range: 12–105) for the CMx group, while it was 60 months (range: 17–107) for the IBR group (*P* = .794). The median age was 50.4 years (range: 25–66) in the CMx group, while in the IBR group, it was 43.4 years (range: 30–86), with a statistically significant difference between the groups (*P* < .001). Patients’ cancers in both groups were predominantly classified as clinically T2 (75.8% in IBR vs 57.6% in CMx, *P* < .001) and T3 (21.2% in IBR vs 37.1% in CMx, *P* < .001) regarding the tumor sizes. Clinically, N1 cases (73.9% vs 54.6%) were more prevalent in the IBR group, while N2 cases (31.8% vs 15.8%) were more common in the CMx group (*P* = .004). According to American Joint Committee on Cancer staging, in both groups, the majority of patients exhibited clinically stage IIB disease, which was more prevalent in the IBR group and statistically significant (69.1% vs 46.2%, *P* < .001). Stage IIIB disease was the least prevalent stage, with a significantly higher incidence in the CMx group (3.9%) compared to the IBR group (1.2%). Stage IIIA cancer constituted 44.6% of the CMx group and 26.1% of the IBR group, demonstrating a statistically significant difference (*P* < .001). The definitive stages of the cases after NACT reflected comparable characteristics. There was no statistically significant difference between the 2 groups regarding the molecular subtypes (*P* = .072). Histologic type, grade, lymphovascular invasion, and pathological multifocality were comparable in both groups, with no significant difference (*P* > .05 for each comparison). The pCR rates were as follows in the CMx group: 14.4% for the breast, 27.3% for the axilla, and 13.6% for both the breast and axilla, while in the IBR group, 18.2% for the breast, 43.6% for the axilla, and 16.3% for both breast and axilla. Although the overall pCR was slightly higher in the IBR group, the difference was not statistically significant (*P* = .375), whereas the axillary pCR was significantly higher in the IBR group (*P* = .001). The primary characteristics of the mastectomy and IBR groups are presented in Table 1.

**Table 1:** Patient’s characteristics.

		CMx alone, n (%)	IBR, n (%)	*P* value
n (%)		132 (44.4)	165 (55.6)	
Age, median (range), yr		50.42 (25–66)	43.36 (30–86)	<.001
Age, yr	<50	68 (51.5)	116 (70.3)	.001
	≥50	64 (48.5)	49 (29.7)	
Median follow-up (range)		61 (12–105)	60 (17–107)	.794
cT stage	T1	2 (1.5)	3 (1.8)	.007
	T2	76 (57.6)	125 (75.8)	
	T3	49 (37.1)	35 (21.2)	
	T4	5 (3.8)	2 (1.2)	
cN stage	N0	11 (8.3)	11 (6.7)	.004
	N1	72 (54.6)	122 (73.9)	
	N2	42 (31.8)	26 (15.8)	
	N3	7 (5.3)	6 (3.6)	
AJCC stage	II B	61 (46.2)	114 (69.1)	.008
	III A	59 (44.6)	43 (26.1)	
	III B	5 (3.9)	2 (1.2)	
	III C	7 (5.3)	6 (3.6)	
ypT stage	ypT0/Tis	25 (18.9)	37 (22.4)	.032
	ypT1	29 (22.1)	51 (30.9)	
	ypT2	53 (40.2)	63 (38.2)	
	ypT3	25 (18.9)	14 (8.5)	
ypN stage	ypN0	36 (27.2)	72 (43.6)	.004
	ypN1	59 (44.7)	61 (37.1)	
	ypN2	30 (22.7)	26 (15.7)	
	ypN3	7 (5.3)	6 (3.6)	
Molecular subtype	HR+/HER2−	63 (47.7)	80 (48.5)	.072
	HR+/HER2+	17 (12.9)	31 (18.8)	
	HER2+	24 (18.2)	35 (21.2)	
	TN	28 (21.2)	19 (11.5)	
Histologic type	IDC	108 (81.7)	130 (78.8)	.342
	ILC	8 (6.1)	16 (9.7)	
	Mixed	7 (5.3)	8 (4.8)	
	Other	9 (6.9)	11 (6.7)	
Histologic grade	1–2	77 (58.3)	95 (57.6)	.093
	3	55 (41.7)	70 (42.4)	
LVI	Yes	56 (42.4)	71 (42.9)	.917
	No	76 (57.6)	94 (57.1)	
Pathologic multifocality	Yes	54 (40.9)	64 (38.8)	.453
	No	78 (59.1)	101 (61.2)	
b-pCR	Yes	19 (14.4)	30 (18.2)	.375
	No	113 (85.6)	135 (71.8)	

AJCC = American Joint Committee on Cancer, b-pCR = breast-pathologic complete response, CMx = conventional mastectomy, HER = human epidermal growth factor receptor, HR = hormone receptor, IBR = immediate breast reconstruction, IDC = invasive ductal carcinoma, ILC = invasive lobular carcinoma, LVI = lympho vascular invasion, TN = triple negative.

### 3.1. Systemic therapy

In terms of treatment, patients in both groups received comparable medical regimens, predominantly consisting of anthracycline, followed by taxane for NACT (78.8% vs 76.4%, *P* = .173). Thirty-eight patients (28.8%) in the CMx group and 62 patients (37.6%) in the IBR group received trastuzumab with or without pertuzumab (*P* = .356). A slightly higher proportion of patients in the IBR group received adjuvant RT (92.7% vs 90.2%, *P* = .54). There were no significant differences between the groups in terms of NACT regimens, adjuvant hormone therapy, and RT (*P* > .05 for all comparisons).

### 3.2. Surgical treatment

Nipple-sparing mastectomy was performed routinely for each patient without involvement of the nipple–areola complex, as confirmed by an intraoperative retroareolar biopsy. If the biopsy indicated tumor presence, we converted the procedure to SSM. In the IBR group, most patients (92.7%) underwent NSM. The procedure was converted to SSM in 12 (7.3%) patients due to tumor presence indicated by the retroareolar biopsy result in 9 patients and final pathology results in 3 patients. All patients underwent ss DTI IBR with silicone prosthesis, and 13 patients in the IBR group underwent contralateral prophylactic mastectomy with reconstruction. The rate of performed sentinel lymph node biopsy, followed by axillary dissection, was not significantly higher in the CMx group compared to the IBR group (69.7% vs 57.6%, *P* = .157). Surgical approaches are shown in Table 2.

**Table 2:** Medical and surgical treatment features.

Variables		CMx alone n = 132 (%)	IBR n = 165 (%)	*P* value
NACT regimen	4 AC + 4 T	104 (78.8)	126 (76.4)	.173
	6 FAC±FEC	17 (12.9)	28 (17.1)	
	Others	11 (8.3)	11 (6.5)	
Adjuvant hormone therapy	Yes	80 (60.6)	111 (67.3)	.134
	No	52 (39.4)	54 (32.7)	
Adjuvant radiotherapy	Yes	119 (90.2)	153 (92.7)	.54
	No	13 (9.8)	12 (7.3)	
Trastuzumab±Pertuzumab in HER2+	Yes	38 (28.8)	62 (37.6)	.356
	No	3 (2.3)	4 (2.4)	
Axillary surgery	SLNB alone	40 (30.3)	70 (42.4)	.157
	SLNB + AD	92 (69.7)	95 (57.6)	
Type of mastectomy	NSM	N/A	153 (92.7)	
	SSM	N/A	12 (7.3)	
	CMx	132	N/A	
Type of reconstruction	Implant with prosthesis	N/A	165	

A = adriamycin, AD = axillary dissection, C = cyclophosphamide, CMx = conventional mastectomy, FAC = fluorouracil–adriamycin–cyclophosphamide, FEC = fluorouracil–epirubicin–cyclophosphamide, HER = human epidermal growth factor receptor, IBR = immediate breast reconstruction, NACT = neoadjuvant chemoptherapy, NSM = nipple-sparing mastectomy, SLNB = sentinel lymph node biopsy, SSM = skin-sparing mastectomy, T = taxane.

### 3.3. Recurrence and survival

The median follow-up duration was 61 months (12–105) for the CMx group and 60 months (17–107) for the IBR group. During this period, local recurrence (LR) was observed in 19 (14.4%) patients in the CMx group and 25 (15.2%) patients in the IBR group, with no statistically significant difference between the groups (*P* = .784). The majority of recurrences manifest as distant metastasis (DM). Although the DM rate was higher in the CMx group, it was not statistically significant (20.5% vs 18.8%, *P* = .844). No statistically significant differences were observed between the CMx and IBR groups in terms of 5-year LRFS (92.4% vs 90.3%, *P* = .653), 5-year DMFS (85.6% vs 85.5%, *P* = .965), 5-year DFS (83.3% vs 80.0%, *P* = .435), disease-specific survival (87.1% vs 87.3%, *P* = .968), and OS (84.8% vs 85.4%, *P* = .948) rates. These findings are given in Table 3.

**Table 3:** Survival rate.

	CMx, n = 132	IBR, n = 165	*P* value
Local recurrence, n	19	25	.784
Distant metastasis, n	27	31	.844
LR + DM, n	13	9	.524
Disease-spesific death, n	17	21	.921
Death, total, n	20	24	.870
LRFS (%)	85.6	84.8	.823
DMFS (%)	79.5	81.2	.762
DFS (%)	75.0	71.5	.675
DSS (%)	87.1	87.3	.968
OS (%)	84.8	85.4	.948
5-yr LRFS (%)	92.4	90.3	.653
5-yr DMFS (%)	85.6	85.5	.965
5-yr DFS (%)	83.3	80.0	.435

CMx = conventional mastectomy, DFS = disease-free survival, DM = distant metastasis, DMFS = distant metastasis-free survival, DSS = disease-spesific survival, IBR = immediate breast reconstruction, LR = local recurrence, LRFS = local recurrence-free survival, OS = overall survival.

The more remarkable and significant factors for the development of recurrence were the cT stage (OR: 2.39 [1.24–4.58], *P* = .009), the cN stage (OR: 2.62 [1.35–5.07], *P* = .004) the ypT stage (OR: 3.22 [1.56–6.66], *P* < .001), and the ypN stage (OR: 2.50 [1.15–5.43], *P* = .020). There was no significant association between other tumor characteristics and recurrence. (*P* > .05 for all comparisons) The factors affecting recurrence are shown in Table 4.

**Table 4:** Univariate and multivariate analysis for recurrence.

Variables		Univariate analysis		Multivariate analysis	
		n = 44	*P* value	OR (95% CI)	*P* value
Age	<50	26 (14.1%)	.737		
	≥50	18 (15.9%)		1.15 (0.60–2.21)	.672
cT stage	T1–2	23 (11.2%)	.012		
	T3–4	21 (23.1%)		2.39 (1.24–4.58)	.009
cN stage	N0–1	24 (11.1%)	.005		
	N2–3	20 (24.7%)		2.62 (1.35–5.07)	.004
ypT stage	ypT0–1	11 (7.7%)	<.001		
	ypT2–3	33 (21.3%)		3.22 (1.56–6.66)	<.001
ypN stage	ypN−	9 (8.3%)	.018		
	ypN+	35 (18.5%)		2.50 (1.15–5.43)	.020
LVI	Positive	21 (12.4%)	.188		
	Negative	23 (18.1%)		1.57 (0.83–2.98)	.169
Ki-67	<20	12 (12.5%)	.489		
	≥20	32 (15.9%)		1.33 (0.65–2.71)	.439
Grade	1–2	22 (12.8%)	.253		
	3	22 (17.6%)		1.46 (0.77–2.77)	.251
HR	Positive	25 (13.1%)	.307		
	Negative	19 (17.9%)		1.45 (0.76–2.78)	.263
HER2	Positive	20 (18.7%)	.175		
	Negative	24 (12.6%)		1.59 (0.83–3.04)	.160
b-pCR	Yes	4 (8.2%)	.189		
	No	40 (16.1%)		2.16 (0.74–6.35)	.150

b-pCR = breast-pathologic complete response, CI = confidence interval, HER = human epidermal growth factor receptor, HR = hormone receptor, LVI = lymphvascular invasion, OR = odds ratio, TN = triple negative.

**Figure 1.**
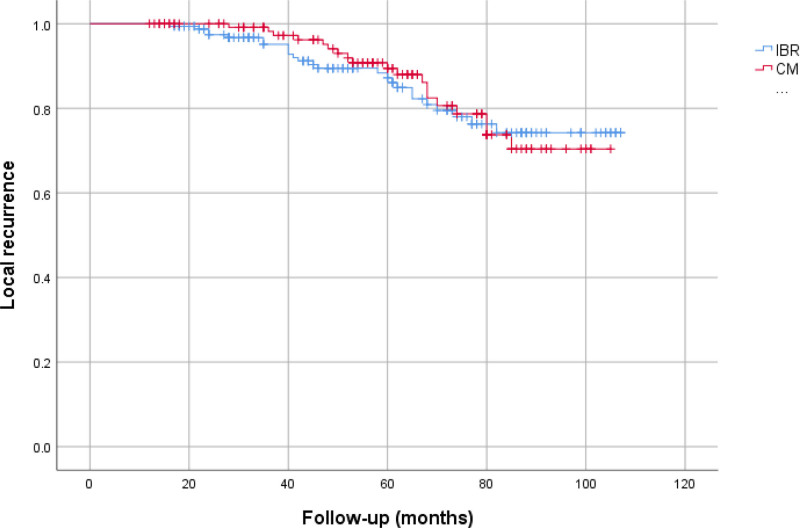
Local recurrence-free survival (IBR, 93.61 ± 2.38 months; CMx, 92.96 ± 2.42 months; *P* = .532). CMx = conventional mastectomy, IBR = immediate breast reconstruction.

**Figure 2.**
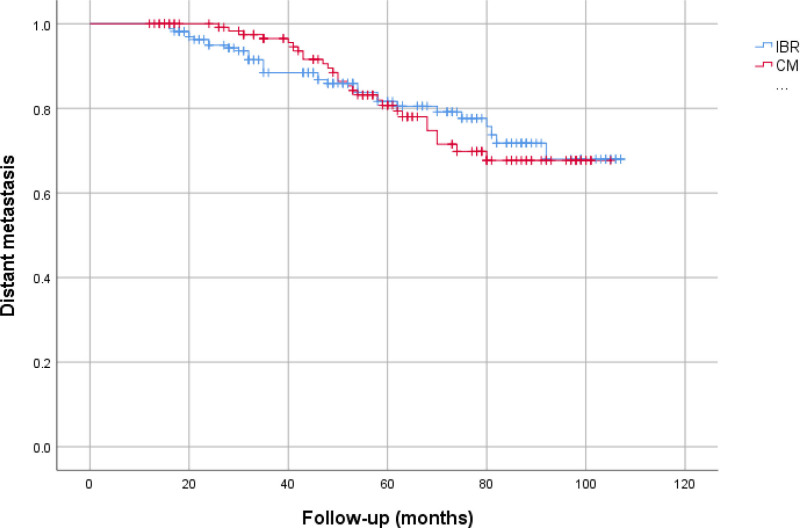
Distant metastasis-free survival (IBR, 90.47 ± 3.59 months, CMx, 88.89 ± 2.62 months, *P* = .703). CMx = conventional mastectomy, IBR = immediate breast reconstruction.

**Figure 3.**
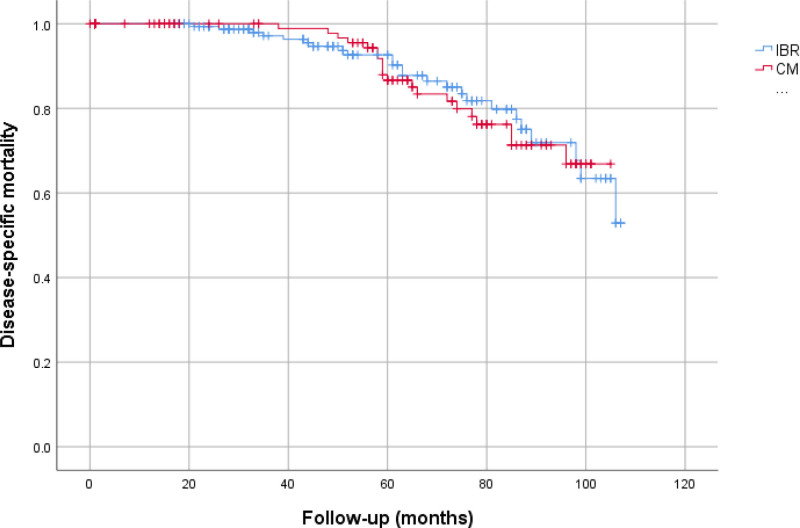
Overall survival (IBR, 94.95 ± 2.17 months, CMx, 93.47 ± 2.24 months, *P* = .916). CMx = conventional mastectomy, IBR = immediate breast reconstruction.

In both groups, the local recurrence rate was most frequently observed in the HER2+ subgroup (20.8% in CMx vs 20% in IBR, *P* = .96). The group with the lowest rate of LR was HR+/HER2− (11.1% in the CMx vs 12.5% in the IBR, *P* = .85). Distant metastasis was most frequently observed in the TN molecular subtype across both groups (39.3% in CMx vs 36.8% in IBR, *P* = .75). Conversely, it was least common in the HR+/HER2− subgroup (11.1%) in the CMx group, and the IBR group, it was least common in the HR+/HER2+ subtype (12.9%). In the CMx group, 17 patients (12.9%), and in the IBR group, 21 patients (12.7%) died due to breast cancer. Table 5 presents information on local recurrence and distant metastasis, considering HER2 receptor status, for both groups. Upon analyzing the survival timelines for both groups, we observed no substantial difference in LRFS (*P* = .532), DMFS (*P* = .703), or OS (*P* = .916) (Figs. 1–3).

**Table 5:** LR and DM rates regarding the molecular subtypes.

	CMx				IBR				*P* *	*P* **	*P* ***
	n	LR n (%)	DM n (%)	Death n (%)	n	LR n (%)	DM n (%)	Death n (%)			
Overall	132	19 (14.4)	27 (20.5)	20 (15.2)	165	25 (15.2)	31 (18.8)	24 (14.5)	.87	.76	.88
HR+/HER2−	63	7 (11.1)	7 (11.1)	5 (7.9)	80	10 (12.5)	13 (16.3)	9 (11.3)	.85	.52	.41
HR+/HER2+	17	3 (17.6)	5 (29.4)	4 (23.5)	31	5 (16.1)	4 (12.9)	4 (12.9)	.92	.32	.24
HER2+	24	5 (20.8)	4 (16.7)	4 (16.7)	35	7 (20)	7 (20)	5 (20)	.96	.75	.75
TNBC	28	4 (14.3)	11 (39.3)	7 (25)	19	3 (15.8)	7 (36.8)	6 (31.6)	.83	.75	.65

CMx = conventional mastectomy, DM = distant metastasis, HER = human epidermal growth factor receptor, HR = hormone receptor, IBR = immediate breast reconstruction, LR = local recurrence, TNBC = triple negative breast cancer.

* Comparison on LR.

** Comparison on DM.

*** Comparison on death.

### 3.4. Complications requiring subsequent surgical procedures and patient satisfaction

There was no difference between groups regarding the reoperation rates for oncologic reasons. In the CMx group, 8 (6.1%) patients required surgery due to local recurrence, and 4 (3%) patients underwent completed axillary dissection. In the IBR group, 12 (7.3%) patients experienced implant loss due to local recurrence. Additionally, 6 patients (3.6%) underwent completion axillary surgery. Patients with distant metastases required no surgical additional interventions. Patients with both local recurrence and distant metastasis received only systemic treatment. In the IBR group, delayed wound healing was the more common observed complication, particularly among diabetic patients and smokers (n = 13, 7.9%). Non-severe infections, resolved with antibiotic therapy, were identified in 12 (7.2%) patients. Spontaneous resolution of seroma occurred in 9 (5.5%) patients, and rippling was observed in 7 (4.2%) patients. None of these complications required surgical intervention. The most frequently observed complication requiring surgical intervention was partial necrosis or ischemia (n = 11, 6.7%). Additionally, 8 (4.8%) patients required re-surgery for capsular contracture, 5 (3%) for severe infection, 2 (1.2%) for extensive hematoma and prosthesis displacement, and 1 (0.6%) for animation deformity. In the IBR group, a total of 20 (12.1%) patients experienced prosthesis loss: 12 (7.3%) due to local recurrence and 8 (4.8%) due to post-surgical and RT complications. In the CMx group, non-oncologic surgical complications included partial ischemia or necrosis in 5 (3.8) patients and postoperative bleeding in 2 (1.5%) patients. Furthermore, 4 (3%) patients received delayed reconstruction using autologous grafts.

When we evaluated the early postoperative, 6-month, and 24-month outcomes of patients who underwent IBR by 2 experienced breast surgery nurses of 160 patients who consented to the evaluation of their photos and participated in the institutional questionnaire, 131 (81.9%) were rated patients’ experience as “excellent” or “good,” and 10 (6.3%) as “poor” or “very poor” in the feedback evaluation.

## 4. Discussion

To our knowledge, this is one of the rare studies in which we have dared to surgically perform a routine IBR procedure in young patients with LABC receiving neoadjuvant therapy and likely to receive adjuvant RT, and additionally to examine complications, oncologic outcomes in this patient group. However, the oncological safety of single-stage IBR in patients with LABC treated with neoadjuvant therapy and who are likely to receive adjuvant RT remains controversial. In this study, we demonstrated that during a median follow-up period of 60 months (range: 12–107), the method of treating LABC patients with NACT, followed by either CMx versus NSM/SSM and single-stage IBR with subsequent RT, is oncologically safe. The single-stage IBR approach was not associated with higher mortality or recurrence rates, and the patients who underwent this approach might experience better physical and psychological well-being. Previous studies have shown that IBR following mastectomy is safe for patients with breast cancer and positively impacts the quality of life, similar to delayed reconstruction while offering better outcomes compared to CMx.^[14,15]^ Yet, given concerns that chemotherapy and RT may increase complication rates, delay adjuvant treatment, and subsequently raise recurrence rates, along with potential difficulties or delays in detecting local recurrences, clinicians preferred still more often 2-stage or delayed reconstruction. However, this study revealed that recurrence rates and survival times were not altered regarding CMx or IBR performed. The 5-year LRFS, 5-year DMFS, 5-year DFS, DFS, and OS rates of the IBR group were 90.3%, 85.5%, 80.0%, 71.5%, and 85.4%, respectively, with no statistically significant difference identified when compared to the CMx group. These results suggest that ss DTI IBR may also be a safe option for patients with LABC planning to receive RT. More importantly, according to our outcomes, recurrence was significantly associated with higher clinical T stage, clinical N stage, pathological N stage, and remarkably higher pathological T stage. We detected that the likelihood of recurrence could increase up to 3.2-fold relative to those with less aggressive tumor features. The risk of having recurrence decreased by 13% in patients with stage T0–1 stage disease compared to patients with stage T2–3.

Reish et al compared patients who underwent DTI reconstruction with and without adjuvant RT. They found statistically higher rates of complications and explantation in the RT group. Additionally, capsular contracture was more prevalent in patients who received RT.^[16]^ Sbitany et al reported that both preoperative and postoperative RT following SSM and IBR with prosthesis led to higher but acceptable complication risks.^[17]^ In a study comparing 62 patients who underwent IBR after NACT with 109 patients who received IBR without chemotherapy, statistically significant differences were for all variables except local recurrence between the groups. Univariate analysis indicated that patients in the NACT group had a 3-fold higher risk of recurrence at a specific time than those in the control group who did not receive chemotherapy (HR: 3.009; 95% CI: 1.349–6.713).^[18]^ While Taqi et al, in their study examining 155 patients with CMx and 112 patients who underwent IBR with a median follow-up period of 50.7 months (range: 0–107), found no differences between the groups in terms of LR, DM, survival rates, and reoperations due to oncological reasons. Among these patients, 77.6% received NACT, and 93.1% received adjuvant RT. The rate of reconstruction with implants was 32.1%.^[19]^ A meta-analysis reviewing 51,731 patients with breast cancer concluded that NACT following IBR does not significantly increase the risk of major or minor complications. The study considered NACT and IBR as safe procedures. Furthermore, the study reported no statistically significant increase in the reoperation rate.^[8]^ In a recent study involving patients with early and advanced-stage breast cancer with axillary lymph node involvement, the authors noted that single-stage IBR is oncologically safe and could be a viable option for suitable patients.^[20]^ In this study period, we initially performed NSM in all patients with no evidence of neoplastic involvement of nipple or areola before NACT, as confirmed by MRI and other radiological imaging. Intraoperatively, surgeons requested a frozen section biopsy on each patient, and in the presence of tumor cells in the biopsy or final pathology, we converted to SSM. Consequently, 92.7% of the patients underwent NSM. To avoid the skin toxicity of RT and facilitate easier detection of potential local recurrences without unnecessary concern, we preferred subpectoral placement of the implants without using ADM or mesh in all patients. The exposed lateral part of the prosthesis was covered by suturing the fascia of the serratus anterior muscle to the pectoralis major muscle. In the guidelines related to RT, the European Society for Radiotherapy and Oncology and the Advisory Committee on Radiation Oncology Practice consensus guidelines recommend exercising caution in high-risk patients due to the possibility of microscopic residual disease in the tissue behind the implant.^[21]^ Indeed, in the early years of implant use, the decision on whether reconstruction would be single-stage or 2-stage was primarily based on the need for post-mastectomy RT. However, advancements in implant technology and radiation therapy equipment, coupled with the increasing experience of radiation oncologists, have led to a reduction in complications associated with the administration of radiation therapy to permanent implants. Therefore, we believe that the most critical parameter in deciding between single-stage or 2-stage reconstruction is not RT but rather the vascularity of the grafts. We prefer single-stage reconstruction in cases with adequate vascularity, while in cases with insufficient circulation, we opt for 2-stage or delayed reconstruction.

Due to oncological reasons, 9.1% of patients in the CMx group and 10.9% in the IBR group underwent re-surgery, which was not statistically significant (*P* = .892). Twelve patients in the IBR group suffered prosthesis removal and needed conversion to CMx due to local recurrence. Flap ischemia and/or necrosis were mainly the causes of additional surgeries not related to oncological reasons, which were addressed through debridement and secondary sutures. During the study, capsulotomy and/or capsulectomy were carried out in 4.8% of patients who developed capsular contracture. In cases of severe infection, drainage or irrigation procedures were conducted to resolve the issue. As a result, in 8 cases, prosthesis loss occurred due to complications, consistent with other studies in the literature.

In our study, the proportion of patients over 50 who underwent CMx and IBR was 48.5% and 29.7%, respectively (*P* = .142). That indicates that reconstruction was predominantly performed on younger patients, while older patients tended to prefer CMx. The fact that 2 (1.2%) patients in the IBR group and 18 (13.6%) patients in the CMx group were aged 65 and over aligns with previous studies reporting that age is a negative predictor for reconstruction, whether by patient choice or surgeon recommendation.^[22,23]^ On the other hand, studies indicate that the rate of post-mastectomy reconstruction has increased in elderly patients in recent years.^[24]^ Although patients in the IBR group were younger and at an earlier stage than those in the CMx group, which may be associated with better survival outcomes, recurrence rates, and complication rates, there was no statistical difference in complication rates between the results.

Although there is no consensus, the single-stage approach offers several advantages, including eliminating the need for a second surgery, shortened recovery times, and a more streamlined treatment process for eligible patients.^[25]^ These developments have been facilitated by improvements in mastectomy techniques, such as skin-sparing and nipple-sparing procedures, which contribute to enhanced aesthetic outcomes and reduced complication rates. Among the 160 participants in the institutional satisfaction questionnaire conducted in both early and late periods, 131 (81.9%) patients rated their experience as excellent or good. In contrast, satisfaction was significantly lower in the population that included patients who had experienced implant loss, with 6.3% of patients rating their experience as poor or very poor.

Our study has several limitations. The single-center retrospective design and the data obtained from a specific patient group may lead to selection bias. In addition, although the tumor-specific characteristics of the patients who underwent both surgical approaches were similar, according to the patient distribution, the fact that our population of patients who underwent IBR consisted of younger patients, their cancers were relatively early stage and responded better to NACT may have affected our oncological results and may lead to conflicting conclusions.

## 5. Conclusion

Single-stage DTI IBR appears to be a safe option for patients with locally advanced breast cancer who receive NACT and are likely to receive subsequent radiotherapy. Supported by oncological safety, low complication rates, and high patient satisfaction, this approach presents a viable reconstructive option for suitable candidates. In addition, breast reconstructions performed without mesh or using ADM can offer oncologically safe and cost-effective options for patients. However, to better establish appropriate indications for ss DTI IBR, it is essential to conduct prospective randomized trials. Such studies will enhance the understanding of the procedure’s efficacy and safety and guide future applications. In this context, continued research and exploration in larger patient populations will help us better understand the potential benefits and limitations of the ss DTI IBR technique.

## Author contributions

**Conceptualization:** Berkay Kilic.

**Data curation:** Berkay Kilic.

**Formal analysis:** Burak Ilhan.

**Investigation:** Berkay Kilic, Burak Ilhan.

**Methodology:** Berkay Kilic.

**Resources:** Berkay Kilic.

**Supervision:** Burak Ilhan.

**Writing – original draft:** Berkay Kilic.

**Writing – review & editing:** Berkay Kilic, Burak Ilhan.
